# Cognitive Risk Stratification Score in Middle-aged and Older Adults With Type 2 Diabetes: A Cross-Sectional Study

**DOI:** 10.1210/clinem/dgaf063

**Published:** 2025-02-04

**Authors:** Jinghua Zhang, Wilson Wai San Tam, Jinhua Lu, Junjie Chen, Joji Kusuyama, Yanhong Dong, Xin Yi Yap, Wentao Zhou, Na Wang, Hui Nan Yeo, Frena Jia Sy Lee, Vivien Xi Wu

**Affiliations:** Alice Lee Centre for Nursing Studies, Yong Loo Lin School of Medicine, National University of Singapore, Centre for Translational Medicine, Singapore 117599, Singapore; Alice Lee Centre for Nursing Studies, Yong Loo Lin School of Medicine, National University of Singapore, Centre for Translational Medicine, Singapore 117599, Singapore; Department of Microbiology and Immunology, Yong Loo Lin School of Medicine, National University of Singapore, Singapore 117545, Singapore; Immunology Translational Research Programme, Yong Loo Lin School of Medicine, National University of Singapore, Singapore 117456, Singapore; Department of Microbiology and Immunology, Yong Loo Lin School of Medicine, National University of Singapore, Singapore 117545, Singapore; Tokyo Medical and Dental University (TMDU), Graduate School of Medicine and Dentistry, Bunkyo City, Tokyo 113-8510, Japan; Alice Lee Centre for Nursing Studies, Yong Loo Lin School of Medicine, National University of Singapore, Centre for Translational Medicine, Singapore 117599, Singapore; Alice Lee Centre for Nursing Studies, Yong Loo Lin School of Medicine, National University of Singapore, Centre for Translational Medicine, Singapore 117599, Singapore; Alice Lee Centre for Nursing Studies, Yong Loo Lin School of Medicine, National University of Singapore, Centre for Translational Medicine, Singapore 117599, Singapore; National Neuroscience Institute, Singapore 308433, Singapore; National University Polyclinics, National University Health System (NUHS), Singapore 609788, Singapore; National University Polyclinics, National University Health System (NUHS), Singapore 609788, Singapore; National University Polyclinics, National University Health System (NUHS), Singapore 609788, Singapore; Alice Lee Centre for Nursing Studies, Yong Loo Lin School of Medicine, National University of Singapore, Centre for Translational Medicine, Singapore 117599, Singapore; NUSMED Healthy Longevity Translational Research Programme, National University of Singapore, Singapore 117456, Singapore

**Keywords:** cognition, cognitive impairment, neuronal function, noninsulin-dependent diabetes mellitus, type 2 diabetes

## Abstract

**Context:**

Cognitive impairment (CI) affects approximately 45% of middle-aged and older adults with type 2 diabetes mellitus (T2DM) globally. Although formal comprehensive neuropsychological tests are the gold standard for diagnosing CI, they are often time-intensive and may not be feasible in primary care.

**Objective:**

This study aimed to develop and validate a novel risk stratification score (RSS) to rapidly and comprehensively predict CI risk among middle-aged and older adults with T2DM, offering a streamlined alternative in clinical practice.

**Methods:**

A cross-sectional study was conducted from July 2023 to February 2024 in a primary care polyclinic in Singapore's western region. Participants aged between 40 and 85 diagnosed with T2DM (n = 150) were included in a convenience sampling. The primary outcome was CI status, which was assessed using formal neuropsychological tests, including the Montreal Cognitive Assessment (MoCA).

**Results:**

CI was identified in 49.3% of participants (n = 74). The RSS, incorporating the MoCA, diastolic blood pressure, and Short Physical Performance Battery, demonstrated excellent discrimination, achieving an area under the receiver operating characteristic curve of 0.802 (*P* < .001). With an optimal cutoff of 0.3, the model showed a sensitivity of 63.5% and specificity of 86.8%, effectively differentiating high- and low-risk CI groups.

**Conclusion:**

RSS in clinical practice, exemplified by the Integrated Metabolic Cognitive Risk Stratification Pathway, is a promising tool for rapid CI risk assessment in primary care. Its robust predictive accuracy and ease of use support its application for early intervention in middle-aged and older adults with T2DM. Future studies should validate its use longitudinally and across diverse populations to enhance generalizability.

Diabetes mellitus is a growing global disease burden, with its prevalence expected to reach 12.2% of the world's population (1) and 14.3% in Singapore among individuals aged 20 to 79 by 2045 (2). Type 2 diabetes mellitus (T2DM) accounts for approximately 90% to 95% of all diabetes cases. Globally, 30% to 70% of patients with T2DM in primary care settings do not meet treatment targets in hemoglobin A1c (HbA1c), blood pressure, and low-density lipoprotein cholesterol lipid control (3). In Singapore, diabetes is the third most prevalent chronic disease managed by polyclinics (4), collectively providing 20% of primary health care (5). T2DM is recognized as 1 of the robust risk factors for cognitive impairment (CI) in the older population (6). The global prevalence of CI among middle-aged and older adults with T2DM averages around 45%, which ranges from 21.8% to 67.5% across different countries (7). Specifically, the mechanism could be contributed by insulin resistance, impaired glucose metabolism, the production of advanced glycosylation end-products, impairment in amyloid beta degradation, increased oxidative stress, and inflammation (8).

Nevertheless, a high proportion of patients with CI remain unnoticed. Formal comprehensive neuropsychological tests are the gold standard for diagnosing CI, covering a wide array of cognitive functions, including memory, attention, executive function, language, and visuospatial skills, but they are time-consuming and resource-intensive. The Montreal Cognitive Assessment (MoCA) is widely used as a brief cognitive screening to detect mild cognitive impairment (MCI) (9). However, it is not as sensitive and comprehensive as formal neuropsychological assessment. Hence, it is essential for a risk stratification tool to comprehensively predict the risk of CI in individuals with T2DM within a brief timeframe.

Risk stratification uses predictive analytics to assess the risk of health conditions, enabling healthcare providers to prioritize care and support early interventions (10). It has been used to predict hospitalization risk in heart failure (11) and dementia in older adults with T2DM (12). Exalto et al (12) identified key predictors of dementia, including microvascular disease, depression, age, and education. However, the data relied on self-reported measures, lacking comprehensive objective measures such as physical examination, biomarkers, or cognitive tests. Our study aims to develop a risk stratification core (RSS) that comprises the risk factors from biomarkers, lifestyle factors, clinical assessment, and self-care ability to predict the risk of CI in T2DM within a reasonable timeframe.

## Materials and Methods

### Study Design, Setting, and Participants

This cross-sectional study was carried out from July 2023 to February 2024. The participants were recruited from a polyclinic in the western region of Singapore that provides 1-stop medical facilities and primary healthcare services (5). Patients with chronic conditions like diabetes, hypertension, hyperlipidemia, and stroke are treated at polyclinics (5). This study follows the Strengthening the Reporting of Observational Studies in Epidemiology (13) reporting guidelines for cross-sectional studies.

**Figure 1.**
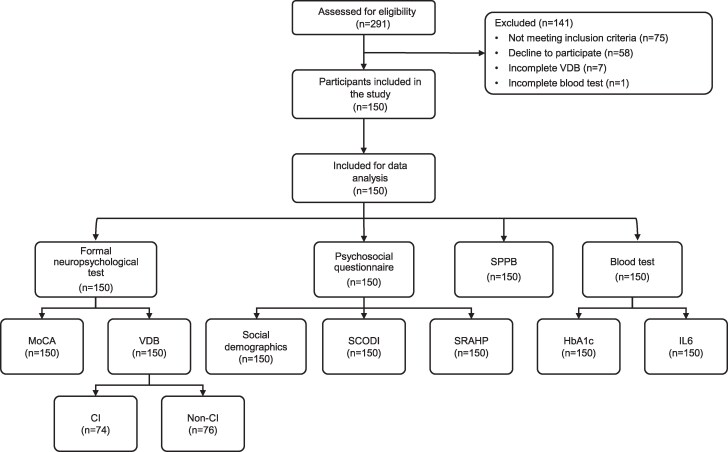
Flowchart for inclusion and exclusion of study participants. Abbreviations: CI, cognitive impairment; MoCA, Montreal Cognitive Assessment; non-CI, noncognitive impairment; SCODI, Self-care of Diabetes Inventory; SPPB, Short Physical Performance Battery; SRAHP, Self-rated Abilities for Health Practices Scale; VDB, vascular dementia battery.

Our research employed a convenience sampling method for participant selection during their polyclinic visits according to the inclusion and exclusion criteria (Fig. 1). The sample size is calculated using the rule of thumb of “15 events per variable” (14) in this logistic regression analysis to ensure enough data for reliable estimation. The prevalence of CI was set as 60% in T2DM according to a previous study (15). Social-demographic and clinical data were collected as the explanatory variables, and it was anticipated that 5 to 6 predicted independent variables would be significantly associated with the CI, such as IL-6 (16), HbA1C (17), MoCA (9), Short Physical Performance Battery (SPPB) (18), and vascular health (blood pressure) (19). Hence, a sample size of 150 participants was calculated.

The inclusion criteria are (1) aged 40 to 85, (2) living in the community, (3) diagnosed with T2DM, (4) literate in English or Mandarin, (5) activities of daily living (ADL) independent, and (6) a score of at least 5 on the SPPB test. The exclusion criteria are (1) severe cognitive (eg, dementia) or psychiatric disorders (eg, schizophrenia, anxiety disorder, or severe depression); (2) severe hearing or vision impairments; (3) terminally ill medical conditions (eg, end-stage cancer) and severe cardiovascular, respiratory (eg, respiratory failure), or orthopedic conditions (eg, frozen shoulder); and (4) pregnant or breastfeeding.

### Variables and Data Sources

#### Outcome variable

CI was defined as failing 1 or more cognitive domains in the formal neuropsychological tests, which include executive function, attention, language, verbal memory, visual memory, visual construction, and visuomotor speed; a domain was considered impaired if the subject failed at least 50% of its tasks (20). The Vascular Dementia Battery was used in this study to categorize subjects into CI and non-CI groups. This formal neuropsychological test had previously been validated with older adults in Singapore (20).

#### Explanatory factors

The explanatory variables to derive the RSS include metabolic health (HbA1c), inflammatory (IL-6), MoCA, SPPB, social-demographic and clinical data, Self-care of Diabetes Inventory (21), and Self-rated Abilities for Health Practices Scale (22). Social-demographic data includes age, sex, ethnicity, religion, marital status, employment status, education level, household type/ownership, income level, living arrangement, and the number of children. Clinical data includes chronic conditions, blood pressure, height, weight, body mass index (BMI), waist-hip ratio (WHR), smoking, alcohol consumption status, and exercise frequency. The Instrumental Activities of Daily Living instrument (23) was used to determine the ADL status during recruiting.

In this study, high HbA1c was operationally defined as having a level of ≥6.5%, a diagnostic criterion for diabetes (24). Similarly, inflammation in diabetes was operationally defined by employing a threshold of 2.3 pg/mL for IL-6 levels (25). IL-6 levels were determined using the Human IL-6 Uncoated ELISA Kit (Catalog # 88-7066-88, RRID: AB_2574995).

### Statistical Analysis

Descriptive statistics were computed for the variables, including frequency distributions, means, and SDs. The chi-square test and independent *t*-tests were generated between CI status and all explanatory variables. Variables that showed association and dependent relationships with CI status were tested via logistic regression. The backward Wald selection method was used to select the best model. The area under the receiver operating characteristic (AUROC) is a performance metric used to evaluate classification models of the RSS, which calculated the cut-off score for distinguishing high-risk and low-risk CI cases. Analyses were conducted at a significance level of *P* < .05 using IBM SPSS (Version 29). Participants were categorized by age, BMI, and WHR. Middle-aged was operationally defined as aged 40 to 60 years old; BMI classifications were normal, overweight, and obese; WHR was classified as normal or central obesity. This study had no missing data in the dataset.

### Integrated Metabolic Cognitive Risk Stratification Pathway

The Integrated Metabolic Cognitive Risk Stratification Pathway (imCRSP) integrates key metabolic and cognitive risk factors into a structured framework derived from a logistic regression model in this study. It provides a practical, evidence-based approach for the early identification of high risk CI in patients with T2DM within primary care settings. By offering clear thresholds and stratification criteria, the imCRSP aids healthcare providers in systematically evaluating patients, facilitating early CI detection and ultimately improving long-term outcomes in T2DM populations.

### Ethics Approval

Ethical approval was obtained from the Domain Specific Review Board (NHG DSRB Ref: 2022/00594), and all subjects signed an informed consent after screening for inclusion and exclusion criteria.

## Results

### Demographic Characteristics

A total of 150 participants were eligible and recruited in the study. The mean age was 65.3 (SD = 8.6); 28% were middle-aged (40-60 years old), while 72% were older adults (>60 years old). The population consisted of 50% female and 50% male, and the ethnic distribution consisted of Chinese, Malay, and Indian of 106 (70.7%), 14 (9.3%), and 27 (18%), respectively, while 2% were of other ethnics, such as Filipinos and Myanmar nationals. Additionally, 24% had no education or primary education level, and 76% had secondary education and above. Furthermore, 40% of the population fell within the healthy weight range, while 60% were overweight or obese, and 19.3% of the participants were smokers (Table 1).

**Table 1.** Demographic and clinical characteristics (n = 150)

Characteristics	n (%)
Sex	
Female	75 (50.0)
Male	75 (50.0)
Race	
Chinese	106 (70.7)
Malay	14 (9.3)
Indian	27 (18.0)
Others	3 (2.0)
Age groups	
Middle-aged adults (40-60 years)	42 (28.0)
Older adults (>60 years)	108 (72.0)
Education	
None	2 (1.3)
Primary	34 (22.7)
Secondary/Institute of Technical Education	67 (44.7)
Preuniversity/Polytechnic	24 (16.0)
University	23 (15.3)
BMI groups	
Healthy weight (18.5-24.9 kg/m^2^)	60 (40.0)
Overweight (25.0-29.9 kg/m^2^)	67 (44.7)
Obese (30 kg/m^2^ or higher)	23 (15.3)
Smoking status	
Yes	29 (19.3)
No	121 (80.7)

Abbreviation: BMI, body mass index.

### Cognitive Impairment

The prevalence rate of CI was 49.3%. The mean age in the CI group was 68.0 (SD = 8.4), while the mean age in the non-CI group was 62.7 (SD = 8.0) (Table 2). The prevalence of impaired cognitive domains was as follows: visual memory 34.7%, verbal memory 19.3%, visuomotor speed 18.7%, visuo-construction 16.7%, executive function 4.7%, language 4.0%, and attention 1.3%.

**Table 2.** Differences in demographics and clinical data according to cognitive function

Characteristics	CI, n = 74	Non-CI, n = 76	*t* or χ^2^	*P*-value	Cohen's d effect size
	Mean (SD) or n (%)	Mean (SD) or n (%)			
Age, y	68.0 (8.4)	62.7 (8.0)	−3.92	<.001	−0.64
Education, y	9.2 (4.1)	11.1 (4.2)	2.95	.004	0.48
IL-6, pg/mL	0.7 (2.4)	0.5 (1.2)	−0.42	.674	−0.07
HbA1c, %	7.5 (1.2)	7.6 (1.4)	0.58	.563	0.10
BMI, kg/m^2^	25.4 (3.8)	27.3 (4.1)	2.80	.006	0.46
Weight, kg	65.9 (12.7)	70.9 (13.1)	2.41	.017	0.39
Hip circumference, cm	100.2 (7.7)	103.0 (8.6)	2.15	.033	0.35
SBP, mmHg	136.9 (16.3)	133.7 (13.8)	−1.29	.198	−0.21
DBP, mmHg	72.9 (8.2)	76.6 (12.3)	2.20	.030	0.36
SPPB score	9.4 (2.0)	10.6 (1.4)	4.31	<.01	0.71
SCODI score	259.5 (63.9)	263.2 (59.4)	0.37	.711	0.06
SRAHP score	82.0 (19.9)	85.5 (17.0)	1.16	.249	0.19
MoCA score	22.3(3.5)	25.5 (2.3)	6.52	<.001	1.07
Age groups, y			7.89	.005	
Middle-aged adults (40-60)	13 (17.6)	29 (38.2)			
Older adults (>60)	61 (82.4)	47 (61.8)			
Sex			<0.001	1.000	
Female	37 (50)	38 (50)			
Male	37 (50)	38 (50)			
BMI groups, kg/m^2^			4.14	.126	
Healthy weight (18.5-24.9)	35 (47.3)	25 (32.9)			
Overweight (25.0-29.9)	31 (41.9)	36 (47.4)			
Obese (30 or higher)	8 (10.8)	15 (19.7)			
WHR groups			0.62	.431	
Normal	11 (14.9)	15 (19.7)			
Central obesity	63 (85.1)	61 (80.3)			
Education level			11.22	.024	
None	2 (2.7)	0 (0)			
Primary	21 (28.4)	13 (17.1)			
Secondary/Institute of Technical Education	34 (45.9)	33(43.3)			
Preuniversity/Polytechnic	12 (16.2)	12(15.8)			
University	5 (6.8)	18(23.7)			
Smoking status			1.24	.265	
Yes	17 (23.0)	12 (15.8)			
No	57 (77.0)	64 (84.2)			

Abbreviations: BMI, body mass index; CI, cognitive impairment; DBP, diastolic blood pressure; HbA1c, hemoglobin A1c; MoCA, Montreal Cognitive Assessment; SBP, systolic blood pressure; SCODI, Self-care of Diabetes Inventory; SPPB, Short Physical Performance Battery; SRAHP, Self-rated Abilities for Health Practices Scale; WHR, waist-hip ratio.

The results of independent *t*-tests showed significant mean differences between the CI and non-CI groups among age (*t* = −3.92, *P* < .001), years of education (*t* = 2.95, *P* = .004), diastolic blood pressure (DBP) (*t* = 2.20, *P* < .030), hip circumference (*t* = 2.15, *P* = .033), BMI (*t* = 2.80, *P* = .006), SPPB (*t* = 4.31, *P* < .001), and MoCA (*t* = 6.52, *P* < .001). There were no significant mean differences between the CI and non-CI groups in IL-6, HbA1c, Self-care of Diabetes Inventory, or Self-rated Abilities for Health Practices Scale (Table 2). The chi-square test showed a significant association between CI status and age groups (chi-square = 7.89, *P* = .005), as well as the education level (above university level or below) (chi-square = 4.75, *P* = .035) (Table 2). However, there was no association between other nominal variables and CI status, such as sex, race, religion, smoking, alcohol consumption, or employment status.

### Logistic Regression

The variables that showed significance in the *t*-test and chi-square test were used to run logistic regression, which includes age, education, weight, BMI, hip circumference, SPPB, DBP, and MoCA. After the elimination, the variables remained in the RSS model encompass DBP [odds ratio (OR) = 0.959, 95% confidence interval 0.923-0.995, *P* = .026], MoCA (OR = 0.690, 95% confidence interval 0.587-0.812, *P* < .001), and SPPB (OR = 0.757, 95% confidence interval 0.585-0.980, *P* = .035) (Table 3).

**Table 3.** Logistic regression analysis for Risk Stratification Score

Predictor variables	OR	SE	95% CI	*P*-value
DBP	0.959	.019	0.923-0.995	.026
MoCA	0.690	.083	0.587-0.812	<.001
SPPB	0.757	.132	0.585-0.980	.035

Abbreviations: CI, confidence interval; DBP, diastolic blood pressure; MoCA, Montreal Cognitive Assessment; OR, odds ratio; SPPB, Short Physical Performance Battery.

### AUROC

#### MoCA cutoff in T2DM

Using the MoCA to predict a low risk of CI demonstrated fair discrimination [area under the curve: 0.776 (0.702-0.850); *P* < .001, Supplementary Fig. S1, Supplementary Data (reference no. 27678204) (26)], with an optimal cut-off score of 25, sensitivity of 73.7%, and specificity of 70.3%. A cut-off score of 25 indicated that participants scoring 25 or higher on the MoCA had a lower risk of CI in T2DM. However, the MoCA's ability to predict a high risk of CI was poor [area under the curve: 0.224 (0.150-0.298); *P* < .001], particularly when MoCA scores were below 25.

#### Cognitive RSS model

**Figure 2.**
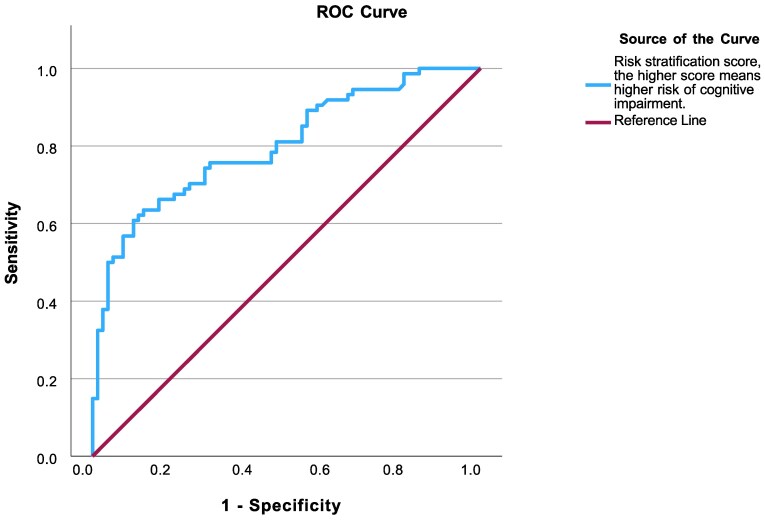
ROC curve of RSS. Fraction of true-positive (sensitivity) and false-positive (1-specificity) results for RSS as a risk marker of cognitive impairment. The calculated area under the curve was 0.802, with 95% confidence interval 0.732 to 0.872 (*P* < .001). Abbreviations: CI, confidence interval; ROC, receiver operating characteristic; RSS, Risk Stratification Score.

The AUROC curve confirmed that the RSS is an excellent model for predicting the risk of CI in T2DM patients [area under the curve: 0.802 (0.732 to 0.872); *P* < .001; Fig. 2], with a good overall model quality [value 0.73, Supplementary Fig. S2, Supplementary Data (reference no. 27678204) (26)]. The AUROC curve enabled us to define a cut-off value for high-risk CI (0.3), with a sensitivity of 63.5% and specificity of 86.8%.

## Discussion

The RSS model, formulated from DBP, MoCA, and SPPB, effectively differentiates high-risk and low-risk CI cases with AUROC among T2DM middle-aged and older adults. Our study demonstrated a large effect size for the MoCA (d = 1.07) between the CI and non-CI groups (Table 2). Medium to large effects were observed for SPPB (d = 0.71) and age (d = −0.64), while small to medium effects were identified for education (d = 0.48), BMI (d = 0.46), weight (d = 0.39), DBP (d = 0.36), and hip circumference (d = 0.35) between the CI and non-CI groups (Table 2). Although HbA1c and IL-6 showed no direct correlation with CI, metabolic and inflammatory markers may influence specific cognitive domains due to factors like demographics, body composition, and vascular health (27). As evidenced in our findings, HbA1c demonstrated a large effect size (d = 1.93), indicating a significant difference between groups with and without impairment in the attention cognitive domain [Supplementary Table S1, Supplementary Data (reference no. 27678204) (26)], while IL-6 showed a large effect size (d = 1.61) for differences in executive function impairment status [Supplementary Table S2, Supplementary Data (reference no. 27678204) (26)].

### DBP and CI

The results indicated that low DBP was independently related to CI. Gupta et al (28) found that DBP appears to be negatively associated with global cognition, executive functions, and memory. Previous studies involving middle-aged and older adults indicated that low DBP could be associated with an increased risk of cognitive decline (29). U-shaped relationships between DBP and cognitive function have been shown previously, which illustrated that the protective window of DBP level was between 90 and 100 mmHg for low risk of Alzheimer's disease (30). Specifically, in participants aged 50 to 70, those with DBP of 70 mmHg or lower had higher rates of cognitive issues compared to those with DBP higher than 70 mmHg (31). Another study suggested older adults with the lowest quartile of DBP equal to or less than 64 mmHg had significantly lower cognitive function scores (32). Our findings showed that lower DBP had higher incidence rates of CI in T2DM, which provided further evidence over the previous studies.

In this study, we observed that those with DBP equal to 70 mmHg or lower had a higher risk of CI among those who scored less than 25 on the MoCA. There are a few plausible reasons that low DBP significantly impacts cognition. First, in older adults with cardiovascular diseases or diabetes, low DBP and aggressive blood pressure reduction raised concerns regarding possible cerebral hypoperfusion, which might potentially result in negative effects on cognitive function, especially in domains of visuospatial and memory recall (31). Second is due to the effect on the small cerebral arterioles that may lead to the development of small cerebral infarctions and diffuse ischemic changes in the periventricular and deep white matter, which can further cause vascular CI by destabilizing neurons and synapses (33). Lastly, low DBP led to vascular endothelial dysfunction in T2DM, reducing nitric oxide or increased inflammation and oxidative stress, further reducing cerebral blood flow and leading to CI (34).

### MoCA and CI

Our study observed significant differences in MoCA scores between the CI and non-CI groups. The MoCA is a brief cognitive screening tool with high sensitivity and specificity for detecting MCI. The normal range for the MoCA is 26 to 30 points (9); however, a cut-off score range of 22 to 25 points has been reviewed for better accuracy in detecting MCI (35). It is a simple 10-minute paper and pencil test that assesses multiple cognitive domains, including memory, language, executive functions, visuospatial skills, calculation, abstraction, attention, concentration, and orientation. Numerous studies have highlighted the necessity for adjusting MoCA cut-off scores based on demographic variables such as age, education/literacy, and race/ethnicity to minimize the misclassification of cognitively normal individuals as impaired (36).

Even though the MoCA alone is not as sensitive and comprehensive as formal neuropsychological assessment for predicting cognitive decline or assessing the risk of future impairment, combining the MoCA with other predictors increases diagnostic accuracy. Shaik et al (37) developed a total risk score to predict cognitive performance in older adults and found that adding the MoCA for those with a positive total risk score improves CI diagnosis in primary care. Another study demonstrated that a combined informatics approach using the MoCA and Functional Activities Questionnaire (a collateral ADL rating) enhanced diagnostic accuracy across the cognitive unimpaired–mild cognitive impaired–dementia continuum relative to cognitive screening alone (38).

### SPPB and CI

The findings showed that the SPPB score, which is a measure of physical performance, has a negative association with CI. Corresponding with the findings, Moon et al (18) reported that a low SPPB score was associated with a 2.22-fold higher risk of CI. Low SPPB scores indicate poor gait speed and muscle loss, which is found to be independently associated with cognitive decline in memory, visuospatial, and construction domains (18). Physical function issues are higher in the sarcopenic population, which is an age-related phenomenon characterized by a dramatic decline in lean body mass over the decades of life (39).

The biological mechanisms accounting for muscle strength decline can arise from skeletal muscle factors, such as loss of muscle mass or changes in muscle architecture and fiber type, but also from neurological factors, such as decreased cortical and spinal excitability, decreased maximal motor unit discharge rate, and slowed nerve conduction (40). Loss of skeletal muscle mass in T2DM results in sarcopenic obesity from malnutrition, insulin resistance, low-grade inflammation, and hormonal changes (41). A study by Levine and Crimmins (16) revealed that chronic inflammation by systemic inflammation and metabolic dysfunction in T2DM can mediate both muscle loss and cognitive function. Our study's findings aligned with this, demonstrating a significant negative correlation between SPPB scores and IL-6 levels (*P* = .006) [Supplementary Table S3, Supplementary Data (reference no. 27678204) (26)]. Elevated IL-6 levels have been linked to obesity and insulin resistance and have been shown to play a role in macrovascular complications in patients with T2DM (34). Sarcopenic obesity was associated with reduced performance in certain cognition domains, including immediate memory and language, and delayed memory in T2DM among those aged 45 and above (42).

### imCRSP

**Figure 3.**
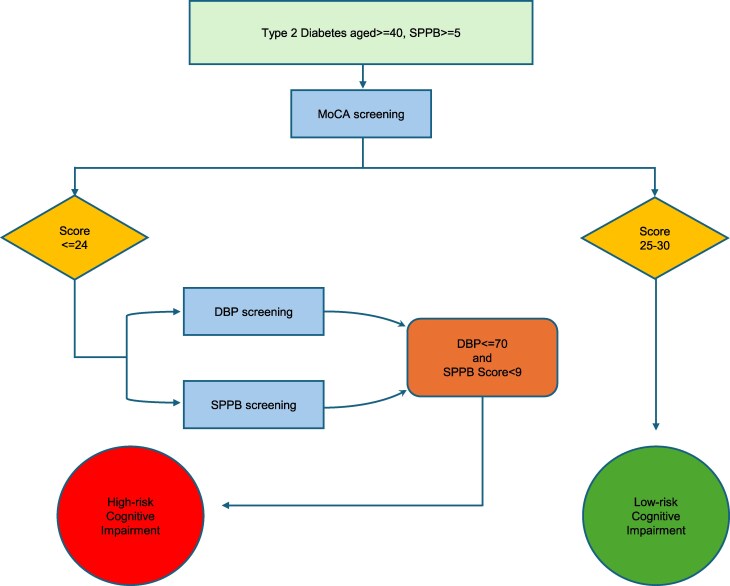
The imCRSP of screening CI risk based on MoCA, DBP, and SPPB. Abbreviations: CI, cognitive impairment; DBP, diastolic blood pressure; imCRSP, Integrated Metabolic Cognitive Risk Stratification Pathway; MoCA, Montreal Cognitive Assessment; SPPB, Short Physical Performance Battery.

The imCRSP was developed based on the population in this study for T2DM, middle-aged and older adults; the observed DBP ranged from 55 to 107 mmHg, MoCA scores ranged from 13 to 30, and SPPB scores ranged from 5 to 12. The imCRSP was validated to high-risk and low-risk CI by comparing the RSS to the established cut-off score of 0.3. Healthcare providers are empowered to effectively stratify patients based on their CI risk levels and activate the appropriate early prevention and management strategies in primary care settings based on assessing the MoCA, DBP, and SPPB. Based on the MoCA cut-off score of 25 in our study, we classified the MoCA score into 2 groups: (1) MoCA scored less than 25 and (2) MoCA scored 25 and above. The imCRSP pathway is more sensitive than the MoCA alone in identifying the risk of CI, especially for the group of MoCA scores less than 25. The imCRSP pathway suggests that those who have a DBP equal to or less than 70 mmHg [Supplementary Table S4, Supplementary Data (reference no. 27678204) (26)] and SPPB score less than 9 [Supplementary Table S5, Supplementary Data (reference no. 27678204) (26)] have a higher risk of developing CI when MoCA scores are less than 25; the calculation is based on the logistic regression and using mean values of SPPB and DBP across different MoCA score levels. There was a lower risk of CI when the MoCA score was 25 and above. The imCRSP indicates the suggested sequence of CI screening according to the DBP, MoCA, and SPPB (Fig. 3).

### Clinical Implication

The imCRSP pathway demonstrates a robust capability in modeling and classifying middle-aged and older adults with T2DM into high-risk and low-risk categories for CI. This approach enables healthcare professionals to regularly screen for CI risk using combined assessments like blood pressure, physical condition (SPPB), and MoCA, allowing for timely preventive measures, such as recommending exercise for at-risk individuals. Early interventions for a healthier lifestyle with adequate exercise and regular assessment of muscle health should be advocated to reduce the risk of CI and disease-induced sarcopenia. Furthermore, the imCRSP emphasized the importance of closely monitoring patients’ DBP when adjusting antihypertensive medications in individuals with T2DM. The pathway suggests that an overly aggressive reduction of DBP may compromise cerebral perfusion (43), thereby increasing the risk of CI. This highlights the need for a balanced approach to managing blood pressure in T2DM patients, considering both cardiovascular and cognitive outcomes.

### Study Strengths and Limitations

The strengths of this study include the use of comprehensive, objective measures such as physical examinations, biomarkers, and formal cognitive tests to predict CI. These objective measures enhance the accuracy and reliability of assessments by reducing subjectivity, providing a robust foundation for early intervention strategies. Additionally, the study introduces the imCRSP pathway, a novel RSS that offers a practical and efficient method for screening CI risk among individuals with T2DM. Meanwhile, the cross-sectional design of this study limits the possibility of establishing a causal relationship between exposure and CI. Future research should adopt a longitudinal approach to track cognitive changes over time, which could provide deeper insights between risk factors and CI in individuals with T2DM. Second, the representativeness of our study sample could be a concern, as participants were recruited from 1 polyclinic in Singapore, which may limit the generalizability of our findings to other populations, such as those from different regions or demographic groups.

## Conclusion

This study developed RSS to facilitate early screening of CI risk in community-dwelling, middle-aged and older adults with T2DM. Its clinical application, demonstrated in the imCRSP, could be a promising screening tool for the early identification of CI risk within primary care settings. Future longitudinal studies will need to assess how cognitive changes evolve over time in individuals with T2DM to enhance RSS.

## Data Availability

Some or all datasets generated during and/or analyzed during the current study are not publicly available but are available from the corresponding author on reasonable request.
